# Telotristat ethyl reverses myxomatous changes in mice mitral valves

**DOI:** 10.3389/fcvm.2022.945672

**Published:** 2022-08-04

**Authors:** Xinmei Wang, Danielle Kuban-Johnston, Pablo Lapuerta, Carla M. R. Lacerda

**Affiliations:** ^1^Department of Bioengineering, Shenyang University, Shenyang, China; ^2^Lexicon Pharmaceuticals, Basking Ridge, NJ, United States; ^3^Department of Chemical Engineering, The University of Texas at Tyler, Tyler, TX, United States

**Keywords:** telotristat ethyl, serotonin, hypertension, myxomatous mitral disease, mouse model

## Abstract

**Rationale:**

Myxomatous mitral valve degeneration is a common pathological manifestation of mitral valve regurgitation, with or without valvular prolapse. In addition to similarities between naturally occurring and serotonergic valve degeneration, an increasing body of evidence has recently suggested that serotonin signaling is a regulator of degenerative valvulopathies. Studies have found that serotonin can be synthesized locally by valvular cells and serotonin receptors in turn may be activated to promote signaling. Recently, telotristat ethyl (TE) has been introduced as a treatment for carcinoid disease, by selectively inhibiting tryptophan hydroxylase 1, the rate-limiting enzyme in peripheral serotonin synthesis. TE provides a unique tool to test inhibition of serotonin synthesis *in vivo*, without impacting brain serotonin, to further confirm the role of local serotonin synthesis on heart valves.

**Objective:**

To confirm the link between serotonin and myxomatous valvular disease *in vivo*.

**Methods and results:**

A hypertension-induced myxomatous mitral valve disease mouse model was employed to test the effect of TE on valvular degeneration. Circulating serotonin and local serotonin in valve tissues were tested by enzyme immunoassay and immunohistochemistry, respectively. TE was administrated in two modes: (1) parallel with angiotensin II (A2); (2) post A2 treatment. Myxomatous changes were successfully recapitulated in hypertensive mice, as determined by ECM remodeling, myofibroblast transformation, and serotonin signaling activation. These changes were at least partially reversed upon TE administration.

**Conclusion:**

This study provides the first evidence of TE as a potential therapeutic for myxomatous mitral disease, either used to prevent or reverse myxomatous degeneration.

## Introduction

The most common mitral valve disorder is mitral regurgitation, often with myxomatous degeneration as the dominant pathology (1). According to the most current Heart Disease and Stroke Statistics update, the prevalence of myxomatous mitral disease is greater than 1.5% in the United States population and dramatically increases with age (1). At the macroscopic level, myxomatous mitral valve leaflets are typically elongated and distorted, leading to prolapse and improper coaptation (2). At the microscopic level, myxomatous mitral valves undergo extracellular matrix (ECM) remodeling and valvular interstitial cell (VIC) phenotype transformation (3).

Valvular homeostasis is maintained by quiescent VICs, the main cell population in normal heart valves (3, 4). In naturally occurring myxomatous mitral valve disease, accompanied or not by valve regurgitation and prolapse, quiescent VICs are transformed to activated VICs, which exhibit increased cell proliferation and enhanced expression of myofibroblastic protein markers, such as alpha-smooth muscle actin (α-SMA), matrix metalloproteinase 1 (MMP1), and transforming growth factor beta 1 (TGFβ1) (3, 5, 6). In addition, ECM remodeling occurs due to increased expression of matrix metalloproteinases (MMPs) and tissue inhibitors of MMPs, collagen degradation, proteoglycan accumulation, and elastin fiber fragmentation (3, 4). Similarly in carcinoid heart disease, patients of carcinoid syndrome develop right heart failure as right-sided heart valves develop a similar pathology (7, 8). The outflow side of the tricuspid and pulmonary valves are known to develop carcinoid plaques, largely composed of highly proliferative myofibroblastic VICs (activated VICs), deposited myxoid ECM rich in collagen and proteoglycans, which together result in valvular regurgitation (9, 10). Carcinoid plaques form more rarely on the left side due to the pulmonary circulation clearing serotonin from arterial blood, which contacts mitral and aortic valves. Despite the differences in location, the cell and molecular changes occurring in myxomatous mitral valve disease and in carcinoid heart disease are evidently similar (8). To further solidify these commonalities, serotonergic valve disease has been found to be inducible by serotonin directly in a murine model (11) or in humans undergoing the Fen-Phen diet, a combination of anorexigen drugs that increase circulating serotonin (fenfluramine and phentermine are both potent inhibitors of serotonin uptake) (12, 13). At the molecular level, serotonergic valve disease is similar to the cases described above, which culminates in potential valve failure (14).

Based on the evidence described above, it has now been demonstrated that serotonin is locally synthesized by mitral valves and regulates myxomatous pathology. Tryptophan hydroxylase 1 (TPH1), the rate-limiting enzyme for peripheral serotonin synthesis, was intensively expressed in myxomatous human and canine mitral valves (15). Additionally, serotonin receptor type 2B (5HTR2b), in company with TPH1, was increased in myxomatous mitral valves, while serotonin transporter was decreased leading its reduced metabolism (16). However, it is still unclear how serotonin circulating in plasma and serum triggers and/or mediates myxomatous mitral disease (17–19). In addition to evidence from *ex vivo* myxomatous mitral valves, other *in vitro* studies have shown that local serotonin in mitral valves can be induced by mechanical stimuli and regulates myxomatous proteins (20, 21).

However, *in vivo* studies to substantiate the role of serotonin regulation on valvulopathies are missing due to the lack of selectivity of TPH1 inhibitors. Recently, telotristat ethyl (TE) has been released for the treatment of carcinoid syndrome. TE is a specific TPH1 inhibitor (22), which does not cross the blood-brain barrier, thus not interfering with serotonin in the brain, synthesized by TPH2. Based on clinical evidence suggesting that TE works effectively to inhibit serotonin synthesis in the gut (22), we expect it to function as an effective inhibitor of serotonin synthesis by heart valves. Thus, here we hypothesized that serotonin synthesis inhibition ameliorates the severity of myxomatous mitral valve disease in hypertensive mice. To test this hypothesis, mice were rendered hypertensive by subcutaneous angiotensin II (A2) delivery with or without TE administration, and parallel groups of normotensive mice served as controls. By studying this mouse model, we aimed to: (1) confirm that mitral myxomatous changes can be induced in hypertensive mice that received a short-term A2 administration; (2) demonstrate that myxomatous changes and high blood pressure correlate with high serotonin; (3) validate the role of TE on myxomatous mitral valves in hypertensive mice; 4) elucidate the link between serotonin and myxomatous valvular disease *in vivo*.

## Materials and methods

### Animals

Wild-type, 6-week old C57BL6J mice of both genders were purchased from Jackson Laboratory (Bar Harbor, ME, United States). Male and female mice were housed separately and maintained under standard conditions within Animal Care Service Facility at Texas Tech University (TTU). Mice in groups of two per cage were maintained under an artificial 12-h dark-light cycle. They were allowed free access to food and water and were acclimated for five. All animal experiments followed TTU Institutional Animal Care and Use Committee (IACUC) approved protocol number T18006.

### ALZET pump preparation

Mini-osmotic pumps, models 2004 or 2006 (both from ALZET, Cupertino, CA, United States) with delivery duration of 4 or 6 weeks respectively, were employed for delivery of A2 or saline. Osmotic pumps were preloaded and primed under sterile conditions following the protocol from the manufacturer. Loading solutions were sterile A2 (Millipore Sigma, Burlington, MA, United States) in 0.9% (w/v) saline (Fisher Scientific, Hampton, NH, United States) or sterile 0.9% saline (Sal). Pre-loaded pumps were primed in sterile 0.9% saline in an incubator at 37°C for at least 40 or 60 h depending on pump model, prior to subcutaneous implantation. In study 1 – prevention mode, mini-osmotic pumps model 2004 filled with A2 delivered 1 μg/kg/min at a flow rate of 0.25 μL/h to achieve 0.8 mg of A2 total per 20 g animal for period of 4 weeks. In study 2 – reversal mode, mini-osmotic pumps model 2006 filled with A2 delivered 1 μg/kg/min at a flow rate of 0.15 μL/h to achieve 1.2 mg of A2 total per 20 g animal for a period of 6 weeks.

### Subcutaneous pump implantation surgery

Right after acclimation, mice underwent implantation of subcutaneous mini-osmotic pumps. Anesthesia chamber was pre-filled with 4% isoflurane (Patterson Veterinary, Greeley, CO, United States) with 2 L/min oxygen flow. After mouse was transferred into chamber, isoflurane was reduced to 2%, and mouse was transferred to a nose cone in ventral recumbence. Eyes were covered by ophthalmic lubricant ointment. Slow release SR Buprenorphine (Wildlife Pharmaceuticals, Windsor, CO, United States) at dose 0.05 mg/kg was administered pre-emptively *via* injection to control pain for 72 h. The skin between the shoulder to the mid-back was shaved and aseptically wiped with 70% alcohol and 4% chlorhexidine gauze sponges for 4 times. Subsequently, animal was immediately transferred to a clean procedural table with a heat pad underneath and covered by a sterile disposable towel. A mid-scapular incision (∼ 10 mm) was made by a sterile blade to open the dorsal skin, and a subcutaneous pocket was created by inserting sterile scissors to fit mini-osmotic pumps. A mini-osmotic pump pre-filled with A2 or saline vehicle was implanted into the pocket. To finalize the surgery procedure, each incision was closed with two 7-mm Reflex wound closure clips (Harvard Apparatus, Holliston, MA, United States). After the surgery, each mouse was transferred to a cage with a clean towel in and a heat pad under to prevent hypothermia and allowed to wake up. All mice were weighed and tattooed on the tail for identification purposes. After surgery, mice were transferred to a new cage and housed in pairs. Mice were maintained under standard conditions at TTU Animal Care Service Facility for 4 or 6 weeks.

### Inducing myxomatous changes and their reversal

Two studies were performed separately and animals were divided into 4 groups (Sal, A2, SalTE, A2TE) in each study. In the first study – prevention mode, ALZET model 2004 pumps preloaded with A2 or saline vehicle (A2 negative control) were surgically implanted into 100 mice (50 males and 50 females) with or without TE administration in parallel for 4 weeks. Briefly, TE was administered daily as described below as the pumps delivered 1 μg/kg/min saline or A2 for 28 days to each animal in pertinent groups. In the second study – reversal mode, ALZET pumps model 2004 or 2006 preloaded with A2 or saline vehicle (A2 negative control) were surgically implanted into 100 mice (50 males and 50 females). Mice implanted with model 2006 pumps received 1 μg/kg/min saline or A2 for 42 days. Mice implanted with model 2004 pumps received 1 μg/kg/min saline or A2 for 28 days followed by a late administration of TE in the last 2 weeks, to reverse previously developed myxomatous changes. Complete experimental details of these two studies are summarized in Table 1.

**TABLE 1:** Experimental design.

Study	Group	Experimental period	Mini-osmotic pump model	Loading solution	Number of Mice			TE administration
					
					Male	Female	Total	
Prevention	Sal	4 weeks	2004	Sal	14	10	24	Not Applied
	SalTE		2004	Sal	10	16	26	Applied for 4 weeks
	A2		2004	A2	14	10	24	Not Applied
	A2TE		2004	A2	12	14	26	Applied for 4 weeks
Reversal	Sal	6 weeks	2006	Sal	12	12	24	Not Applied
	SalTE		2004	Sal	10	14	24	Applied in the last 2 weeks
	A2		2006	A2	14	12	26	Not Applied
	A2TE		2004	A2	14	12	26	Applied in the last 2 weeks

Telotristat ethyl was provided by Lexicon Pharmaceuticals (Basking Ridge, NJ, United States). TE was prepared as a suspension in 0.25 % (w/v) methylcellulose (Spectrum, New Brunswick, NJ, United States) to a final concentration of 0.24 g/mL TE. Each mouse was fed 25 μL TE solution once each day by oral gavage to achieve 300 mg/kg/day, a dose at which dose peripheral serotonin was significantly reduced in mice guts, without inducing side effects.^2^ TE was administered either in parallel or post-A2 treatment until the day before euthanasia.

### Blood pressure *in vivo* measurement

Blood pressure measurements were conducted using the CODA non-invasive blood pressure system (Kent Scientific, Torrington, CT, United States) every 4 days over the complete experimental period. The procedure followed the owner’s manual from Kent Scientific. A CODA controller was connected to a laptop installed with CODA software (CODA 4.1), and a warming platform was preset to 38 *^o^*C. After mice were properly placed in pre-warmed holders, their tails entered the CODA cuff assembly with the occlusion cuff close to tail base and the volume pressure recording (VPR) sensor distal to the O-cuff. Both O-cuff and VPR sensor were connected to the CODA controller. Data tables were created by the system containing systolic, diastolic, and mean blood pressures. Statistical differences on blood pressure among treatments were assessed by multiple comparison tests following one-way ANOVA and *p* < 0.05 were considered significant.

### Exsanguination and heart collection

At the end of *in vivo* treatments (4 or 6 weeks), mice underwent carbon dioxide asphyxiation. After mice ceased breathing, they were weighed and transferred to an operation bench. The death was confirmed by cervical dislocation. Subsequently, blood was collected through the abdominal aorta and hearts were collected through a thoracic incision. Blood samples were immediately supplemented with 0.1% ethylenediaminetetraacetic acid (Fisher Scientific) and stored at −20°C for future serotonin enzyme-linked immunosorbent assay. Hearts were washed at least 3 times in phosphate-buffered saline [PBS, 137 mM sodium chloride, 2.7 mM potassium chloride, 4.3 mM sodium phosphate dibasic and 1.46 mM potassium phosphate monobasic (all from Fisher Scientific)] supplemented with 0.1% ethylenediaminetetraacetic acid, followed by fixation in 4% formaldehyde (Fisher Scientific). Paired Student’s *t*-test was performed to assess the significant differences on animal weight changes with age. *P* < 0.05 were considered significant.

### Circulating serotonin quantification

The total concentration of serotonin circulating in blood was determined by serotonin isolation from blood proteins followed by a serotonin enzyme-linked immunosorbent assay (Enzo Life Science, Farmingdale, NY, United States). Briefly, blood samples were centrifuged, then precipitated in 9 volumes of ethanol at −80°C overnight to remove hemoglobin and other proteins after complete cell lysis (23). Serotonin was enriched in the supernatant ethanol layer and vacuum concentrated. Enzyme-linked immunosorbent assay was performed following the manufacturer’s protocol. The average and standard deviation in each group were calculated from eight biological replicates. Statistical differences on circulating serotonin among treatments were assessed by multiple comparison tests following one-way ANOVA, and *p* < 0.05 were considered significant.

### Histology and histological staining

#### Histological slide preparation

Mouse hearts were symmetrically bisected across the center of the ventricles. After fixation in formaldehyde, dehydration, clearing and paraffin infiltration, heart sections were paraffin-embedded in tissue cassettes with left ventricles next to each other. Tissue-embedded paraffin blocks were sliced into 4 μm slices by a rotary microtome and slices placed onto poly-L-lysine coated glass slides (Fisher Scientific). Sections were air dried overnight prior to deparaffinization in xylenes and subsequent rehydration prior to staining.

#### Modified russell-movat pentachrome stain

Mitral valve sections were stained by a Movat-Russell modified pentachrome stain kit (American MasterTech, Lodi, CA, United States). The staining procedure followed the protocol recommended by the manufacturer. Elastic fibers and nuclei were stained black and dark purple, respectively. Polysaccharides, muscle and collagen were stained in sequence by alcian blue, the combination of crocein scarlet and acidic fuchsine, and alcoholic saffron in greenish blue, red, and yellow, respectively. Sections were allowed to air dry and mounted with Permount mounting medium (Fisher Scientific) and cover glasses. Bright-field images were acquired using a color camera (DFC295) of Leica DMI6000 B microsystem (Leica Microsystems, Buffalo Grove, IL, United States). Valve thicknesses were measured at the base of the leaflets on histological sections using a scale bar in LAS X microscope software (Leica Microsystems). Average values and standard deviations of thickness measurement were calculated from 26 images from at least 6 biological replicates. Statistical differences on valve thickness among treatments were assessed by multiple comparison tests following one-way ANOVA, and *p* < 0.05 were considered significant.

#### Immunohistochemistry

To investigate differences in expression of protein markers on mitral valves receiving different treatments, Immunohistochemistry (IHC) was performed using a commercial ready-to-use kit (BioVision, Milpitas, CA, United States). The protocol provided by the manufacturer was followed. First, peroxidase block was applied to tissue sections for 10 min. After 10 s wash in water, sections were immersed in pre-boiled sodium citrate buffer (10 mM sodium citrate, 0.05% Tween 20 (both from Fisher Scientific), pH 6.0) and immediately transferred to a pre-heated steamer. The requirement of heat-induced antigen retrieval step depended on the primary antibody applied later. In this study, antigen retrieval was employed in the staining of 5HTR2b, TGFβ1, and MMP1. Sections were then cooled in cold water and incubated with protein blocking solution for 10 min. Primary antibodies were applied to tissue sections and incubated for 30–60 min without drying. Primary antibodies employed were mouse monoclonal anti-TPH (Millipore Sigma) at a final concentration of 2 μg/mL, mouse monoclonal anti-smooth muscle actin (Fisher Scientific) at a final concentration of 1.25 μg/mL, rabbit polyclonal anti-5HTR2b (Novus Biologicals, Centennial, CO, United States) at a final concentration of 2.4 μg/mL, rabbit polyclonal anti-TGFβ1 (Abcam, Cambridge, United Kingdom) at a final concentration of 5 μg/mL, rabbit polyclonal anti-MMP1 (Invitrogen, Carlsbad, CA, United States) at a final concentration of 0.92 μg/mL. After primary antibody binding, another 30-min incubation with HRP polymer was performed, followed by washes in PBS and water in sequence. Positive expression of proteins was visualized in a dark brown color after a 10 min reaction with diaminobenzidine chromogen. Lastly, nuclei were labeled by hematoxylin (Electron Microscopy Sciences, Hatfield, PA, United States) in dark purple, and sections were washed in water for 20 min for better coloration. Sections were mounted with Fluoromount-G hydrophilic mounting medium (Southern Biotech, Birmingham, AL, United States) and cover glasses after air drying. Bright-field images were acquired as above. For the quantitative evaluation of each protein marker, at least 4 biological replicates in each treatment group were tested. Protein expression was quantified in MATLAB R2018b (MathWorks, Natick, MA, United States), where the total stained area in pixels was measured from at least 4 biological replicates after background and noise were removed by thresholding. Significant differences in protein expression were assessed by Kruskal-Wallis and multiple comparison tests. *P* < 0.05 were considered significant.

## Results

### All mice were initially normotensive

In the prevention study, average weights of animals increased from 21.5 3.5 g to 22.6 3.7 g in 4 weeks, while in the reversal study, average weights increased from 22.0 2.8 g to 25.0 3.2 g in 6 weeks. In both cases, weights of males were always higher than those of females, with no significant differences observed across treatments (Supplementary Figures 1A,B).

**FIGURE 1:**
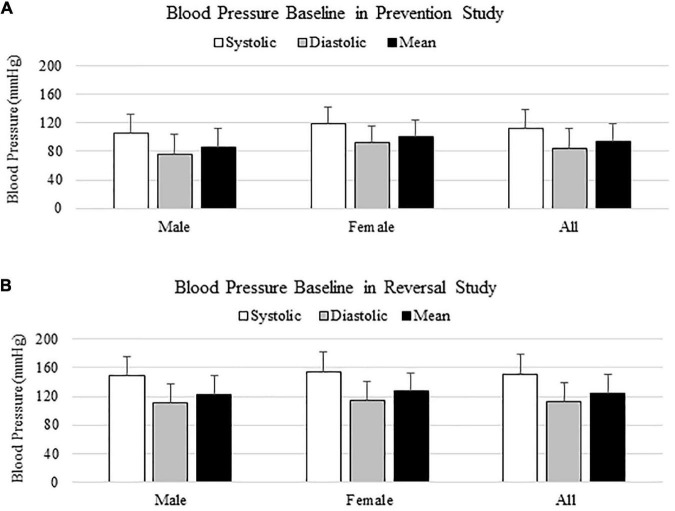
Blood pressure baseline in prevention **(A)** and reversal **(B)** studies.

In both prevention and reversal studies, blood pressure baseline was measured from 100 animals the day before surgeries, and animals were all normotensive initially (Figures 1A,B). Blood pressure baselines in reversal study were higher than those from prevention study, due to a different size of occlusion cuff used in reversal study. In both cases, there was no significant difference on blood pressure baseline between males and females, as shown on Figures 1A,B.

### Hypertension was successfully induced by subcutaneous angiotensin II delivery in mice

Systolic, diastolic and mean blood pressures of each animal were measured every 4 days over the entire experimental periods of both studies. Since paired systolic, diastolic and mean blood pressures showed the same trends, only systolic blood pressures (SBP) are reported here and were used to assess differences across treatments.

**FIGURE 2:**
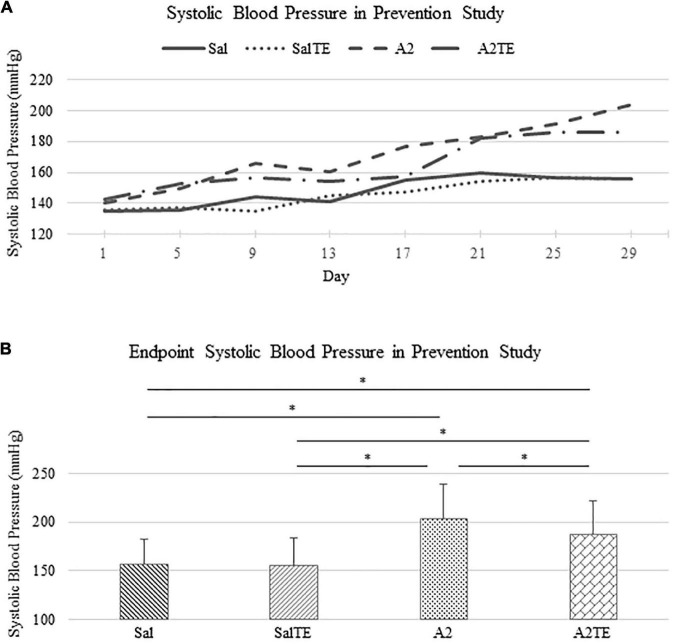
**(A)** Systolic blood pressure changes with time in hypertensive and normotensive mice in prevention study. **(B)** Endpoint (Day 29) systolic blood pressure comparison across treatments. Asterisk indicates significant difference (*p* < 0.05).

In the prevention study, SBPs from Sal and SalTE groups overlaid throughout the entire experimental period, and were much lower than SBPs from A2-treated groups on each measurement date (Figure 2A). However, the parallel administration of A2TE ceased the increase in blood pressure after day 21. This demonstrates that A2 dramatically elevated blood pressure from 149.6 35.6 mmHg to 203.8 34.6 mmHg in mice, and sustained TE administration seemed to reverse A2-induced hypertension, as indicated by a less increase in blood pressure from 142.7 32.2 mmHg to 185.8 36.7 mmHg in 4 weeks. To further evaluate the effects of A2 and/or TE on blood pressure, blood pressures from final measurements were compared across treatments, as shown in Figure 2B. Final day blood pressures from angiotensin groups (A2 and A2TE) were significantly higher than those from saline groups (Sal and SalTE). Moreover, TE decreased blood pressure in A2TE but not SalTE. The main findings are 1) A2 significantly induced hypertension in mice within 4 weeks; 2) TE reversed A2-induced hypertension in mice, without effects in normotensive mice.

**FIGURE 3:**
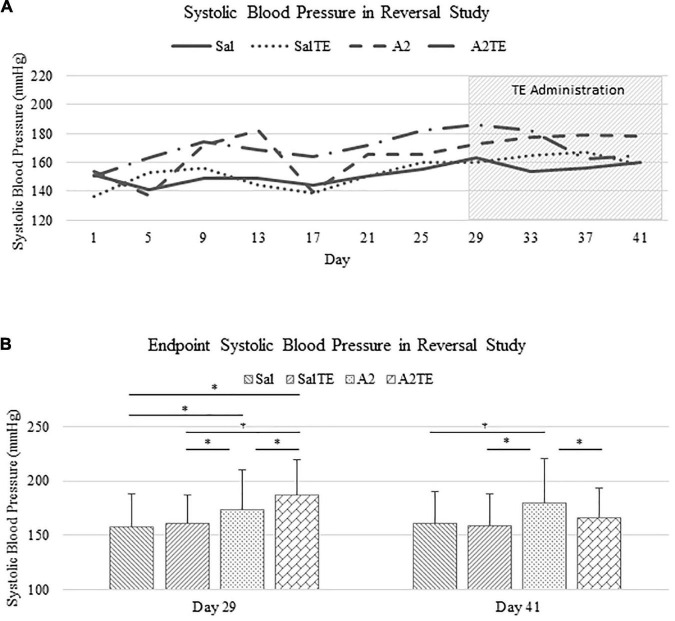
**(A)** Systolic blood pressure changes with time in hypertensive and normotensive mice in reversal study. **(B)** Endpoint (Day 29 and 41) systolic blood pressure comparison across treatments. Asterisk indicates significant difference (*p* < 0.05).

In the reversal study, TE was administered in the last 2 weeks of the total 6 weeks of A2 treatment. Consistently with the observations from the prevention study, A2 largely induced high blood pressure from 153.9 37.0 mmHg to 178.4 42.6 mmHg, which was reversed by TE supplementation, where blood pressure was elevated from 150.2 32.0 mmHg to 164.9 28.8 mmHg (Figure 3A). On the other hand, TE administration did not result in differences in blood pressure among saline groups (Figure 3A). Again, endpoint blood pressures before and after TE administration (Day 29 and Day 41) were compared across treatments. Before TE administration (Day 29), blood pressures in angiotensin groups (A2 and A2TE) were significantly higher than those from saline groups (Sal and SalTE) (Figure 3B). After 2 weeks along with TE administration (Day 41), blood pressure was continuously increased by A2, but this change was prevented by TE (Figure 3B). However, TE administration did not cause a change in saline group (SalTE). By the end of the experiment, blood pressures from A2 group were significantly higher than those from the other 3 groups, indicating an impact of TE on A2-induced high blood pressure.

These findings demonstrate that A2 continuously increased blood pressure in mice for 6 weeks, but the resulting high blood pressure was significantly reversed by TE administration. Data from both studies suggested that A2 successfully induced hypertension in mice and TE counteracted this effect in either parallel or late administration.

### Angiotensin II and telotristat ethyl synergistically reduce circulating serotonin in mice

**FIGURE 4:**
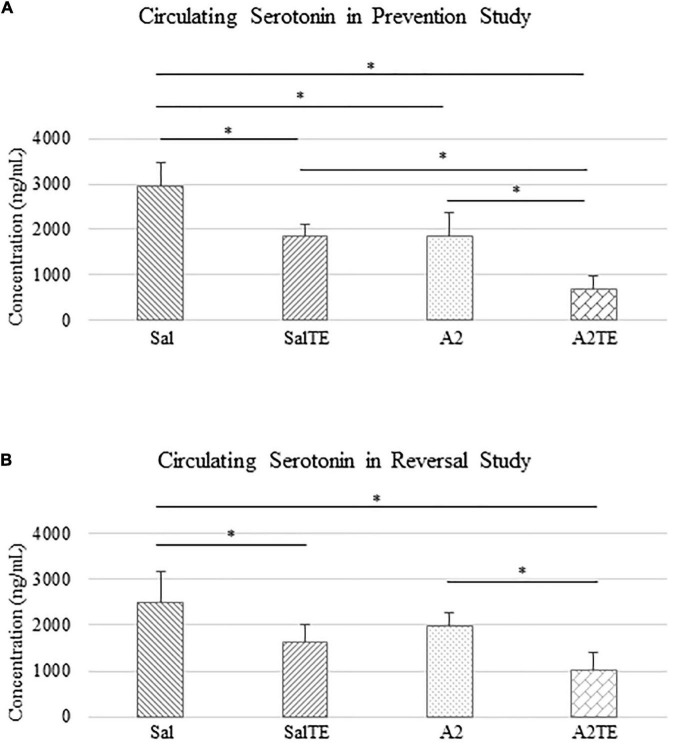
Circulating serotonin level comparison across treatments in prevention **(A)** and reversal **(B)** studies. Asterisk indicates significant difference (*p* < 0.05).

Circulating serotonin levels were compared across treatments. In the prevention study, A2 significantly reduced circulating serotonin in mice, compared to Sal group that served as normotensive control (Figure 4A). Concentrations of circulating serotonin in TE-treated mice were significantly lower than those in the corresponding Sal or A2 groups (Figure 4A), as expected. However, the inhibitory effect of TE on serotonin was higher in the presence of A2 (Figure 4A). TE reduced circulating serotonin from 2952.51 to 1862.00 ng/mL on average in normotensive mice, and the inhibition efficiency is 36.94%. On the other hand, TE decreased 62.46% circulating serotonin in hypertensive mice, with average levels of 1838.82 and 690.33 ng/mL, respectively.

Similar results of circulating serotonin concentrations were observed in reversal study, where TE was administered following the treatment with A2 or Sal. A2 reduced circulating serotonin after 6 weeks (Figure 4B), although not significantly. The 2-week administration of TE significantly reduced serotonin levels in blood compared to those in corresponding Sal and A2 (Figure 4B). Moreover, the lowest concentration of serotonin appeared in A2TE as before (Figure 4B). These data suggested that longer A2 treatment slightly reduced serotonin level, while TE always effectively inhibited circulating serotonin. Moreover, this inhibitory effect was maximized in combination with A2. Circulating serotonin was reduced by TE from 2497.15 to 1634.61 ng/mL in normotensive mice and from 1978.64 to 1016.05 ng/mL in hypertensive mice, resulting 34.54 and 48.65% reductions, respectively.

### Myxomatous changes on mitral valves were observed in hypertensive mice, and reversed by a parallel telotristat ethyl administration

**FIGURE 5:**
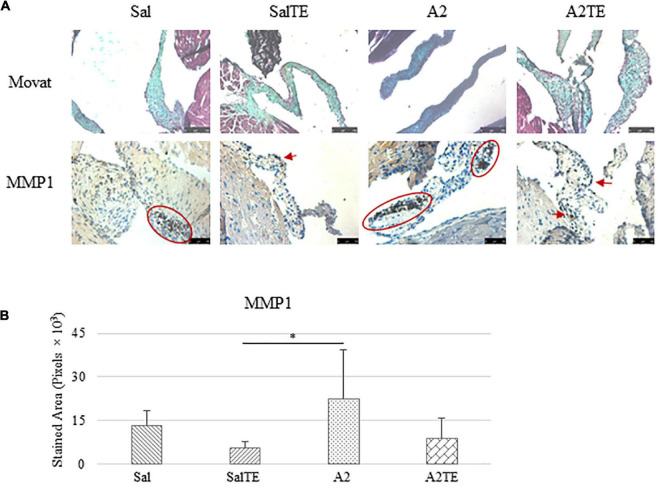
**(A)** Investigation of mitral valve ECM remodeling by Movat stain and MMP1 IHC in prevention study. Major positive IHC stains were highlighted by circles and arrows. Scale bar on Movat images: 100 μm. Scale bar on IHC images: 50 μm. **(B)** Quantification of MMP1 expression determined by total stained area in pixels that measured from 4 biological replicates in MATLAB. Asterisk indicates significant difference (*p* < 0.05).

**FIGURE 6:**
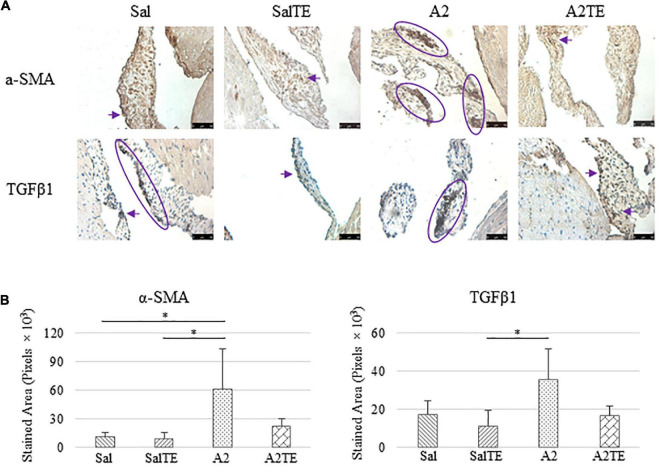
**(A)** Investigation of mitral valve VIC activation by IHC examinations on α-SMA and TGFβ1 in prevention study. Major positive IHC stains were highlighted by circles and arrows. Scale bar: 50 μm. **(B)** Quantification of their expression determined by total stained area in pixels that measured from 4 biological replicates in MATLAB. Asterisk indicates significant difference (*p* < 0.05).

Myxomatous remodeling was evaluated on Movat stains and IHC of myxomatous markers. ECM disorganization was assessed by Movat stains, where collagen, proteoglycans, and elastic fibers were colored in yellow, greenish blue, and black, respectively. The most easily observed ECM component is proteoglycan, as indicated by greenish blue tint throughout the sections (Figure 5A). In addition, black elastic fibers dominated the atrialis layer facing the left atrium. A much more condensed blue tint appeared in mitral valves of A2-treated mice, indicating that proteoglycan accumulation was induced by A2 (Figure 6A), with less yellow (collagen fibers). Whereas, this proteoglycan overproduction combined with collagen fragmentation was not observed in the presence of TE (Figure 6A). Mitral valve thickening did not occur in short-term (4 week prevention study) A2 supplementation (Supplementary Figure 2A).

**FIGURE 7:**
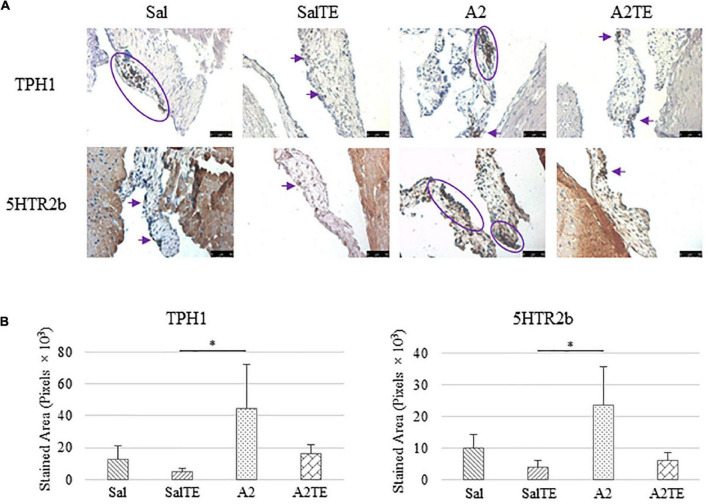
**(A)** Investigation of local serotonin signaling in mitral valve by IHC examinations on TPH1 and 5HTR2b in prevention study. Major positive IHC stains were highlighted by circles and arrows. Scale bar: 50 μm. **(B)** Quantification of their expression determined by total stained area in pixels that measured from 4 biological replicates in MATLAB. Asterisk indicates significant difference (*p* < 0.05).

Expression of five myxomatous markers were assessed by IHC in mitral valves from the same animals employed for Movat stains. Positive expression of protein markers was stained in dark brown, indicating affected regions. In general, excess MMP1 was produced by mitral valves that received A2 (Figures 5A,B). Parallel treatment with TE lowered MMP1 level compared to corresponding Sal or A2 specimens (Figures 5A,B). Activated phenotype transformation was significantly enhanced in mitral valves in hypertensive mice, as suggested by plaques of strong positive α-SMA stain (Figures 6A,B). As expected, parallel administration of TE reduced A2-induced cell activation. TGFβ1 showed the same trend as α-SMA, with its expression significantly increased in hypertensive mice but reversed by TE (Figures 6A,B). TPH1 and 5HTR2b expression levels were highly increased in hypertensive mice, and diminished by TE administration (Figures 7A,B). Moreover, their expression was also lowered by TE in normotensive mice. Based on these observations, the prevention study uncovered that ECM proteoglycans and myxomatous markers (MMP1, α-SMA, TGFβ1, TPH1, and 5HTR2b) were overproduced in mitral valves of hypertensive mice, and these changes were reversed by TE.

### Myxomatous changes on mitral valves were observed in hypertensive mice, and reversed by a late telotristat ethyl administration

**FIGURE 8:**
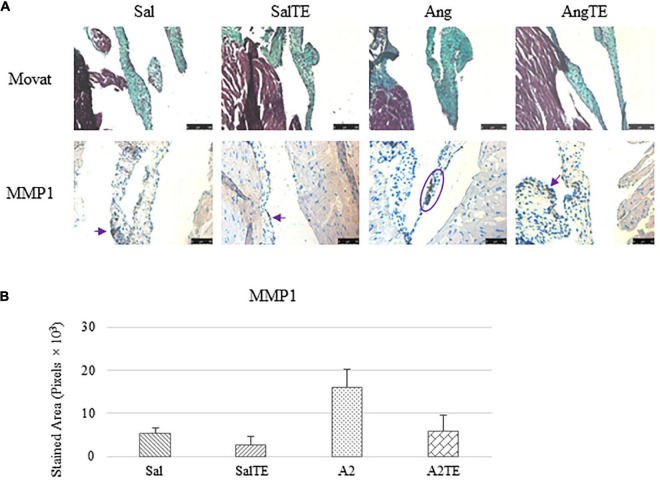
**(A)** Investigation of mitral valve ECM remodeling by Movat stain and MMP1 IHC in reversal study. Major positive IHC stains were highlighted by circles and arrows. Scale bar on Movat images: 100 μm. Scale bar on IHC images: 50 μm. **(B)** Quantification of MMP1 expression was determined by total stained area in pixels that measured from 4 biological replicates in MATLAB. Asterisk indicates significant difference (*p* < 0.05).

**FIGURE 9:**
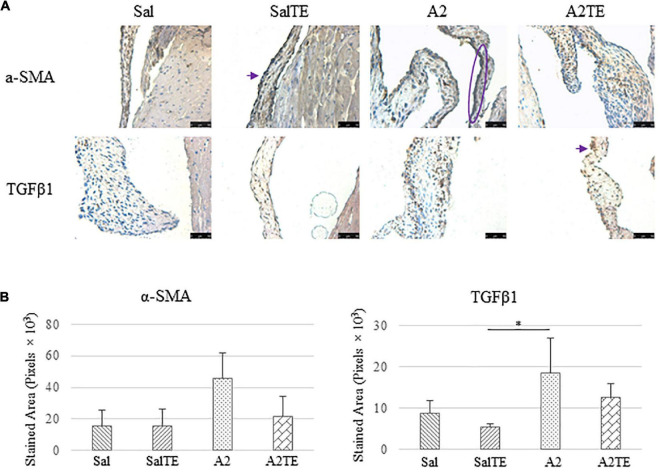
**(A)** Investigation of mitral valve VIC activation by IHC examinations on α-SMA and TGFβ1 in reversal study. Major positive IHC stains were highlighted by circles and arrows. Scale bar: 50 μm. **(B)** Quantification of their expression determined by total stained area in pixels that measured from 4 biological replicates in MATLAB. Asterisk indicates significant difference (*p* < 0.05).

**FIGURE 10:**
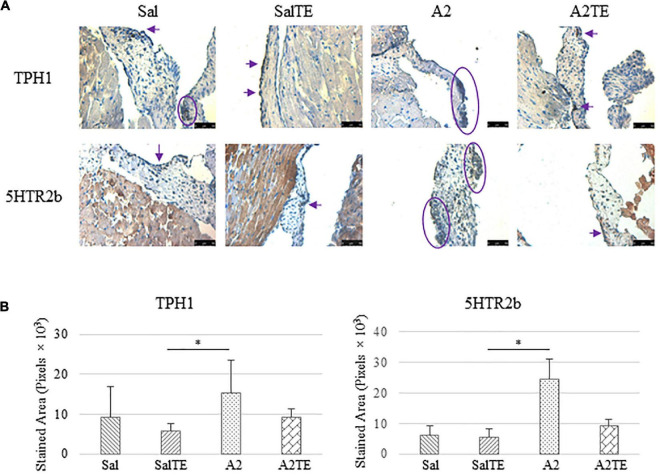
**(A)** Investigation of local serotonin signaling in mitral valve by IHC examinations on TPH1 and 5HTR2b in reversal study. Major positive IHC stains were highlighted by circles and arrows. Scale bar: 50 μm. **(B)** Quantification of their expression determined by total stained area in pixels that measured from 4 biological replicates in MATLAB. Asterisk indicates significant difference (*p* < 0.05).

Consistently with the observations from the prevention study, the reversal study showed that proteoglycans accumulated in A2-treated mitral valves, as suggested by a higher density of greenish blue stain (Figure 8A), and this was effectively reversed by a 2-week late administration of TE. As before, MMP1, α-SMA, TGFβ1, TPH1, and 5HTR2b were assessed in mitral valves obtained from the reversal study. In general, they all presented similar trends as those observed from the prevention study. MMP1 (Figures 8A,B) expression was higher in hypertensive than normotensive mice, and in both cases, TE decreased MMP1 expression. α-SMA and TGFβ1 (Figures 9A,B) levels were significantly increased by A2, and these trends were reversed by TE. Also in agreement with the prevention study, TPH1 and 5HTR2b (Figures 10A,B) expression levels in mitral valves were always higher in hypertensive mice, but decreased upon TE administration. In addition to the significant changes on myxomatous proteins, mitral valves were significantly thickened by A2, compared to those from normotensive animals (Supplementary Figure 2B). The major finding from the reversal study is that ECM proteoglycans and all protein markers were overproduced in mitral valves of hypertensive mice, and these changes were at least partially reversed by a 2-week late TE administration.

## Discussion

Angiotensin II has been known to induce hypertension in mice and its effect has been validated in several studies (24, 25). Our goal was to employ this model as an inducer of myxomatous disease and use it as a tool to uncover the role of serotonin in myxomatous mitral disease. Previously, hypertension was been successfully induced *via* chronic systemic A2 infusion in mice with mitral valves presenting myxomatous changes (26). Additional indirect evidence suggested that A2 is a contributor to the progress of myxomatous mitral disease (27, 28). The SBP reported for C57BL/6J at age 8–14 weeks, is 120 mmHg, (29) which is comparable to our experimental results.

It has been demonstrated that blood pressure increases with age in mice, as it does in humans (30–32). In both studies presented here, TE administration seemed to curb the effect of A2 on blood pressure, while maintaining the blood pressure of normotensive animals. Moreover, females were more responsive to TE effect on blood pressure reduction, which might be a consequence of higher dose of TE due to lower body weight. Several lines of evidence demonstrated that high levels of circulating serotonin upregulates blood pressure, due to its contractile effect on vascular smooth muscle cells (33, 34). This could explain why lower serotonin (due to TE administration) counteracted A2-induced hypertension in mice. However, this TE-induced blood pressure drop was not observed in humans, when TE was studied as a treatment for carcinoid syndrome (22).

Currently, there is limited evidence showing the alteration of circulating serotonin level in hypertensive subjects. Blood samples collected from hypertensive and normotensive human showed no significant difference on serotonin levels (35). Additional studies implied that the correlation between blood serotonin and hypertension still remains controversial (36, 37). In our work, serotonin was downregulated by TE administration in both hypertensive and normotensive mice. The decrease in serotonin in hypertensive mice may be due to the interplay between serotonin and A2, which might not happen to the same extent in clinical systemic hypertension. It has been suggested that serotonin level was reduced in platelets while elevated in plasma in hypertensive mice, although the total content in the blood remained constant (38). In agreement with this, human studies proposed that platelet serotonin level decreased with hypertension due to a reduced uptake rate by serotonin transporter (39, 40). A2 administration in rodents was previously reported to significantly promote the level of 5-hydroxyindoleacetic acid, a serotonin metabolite (41), indicating that A2 accelerates serotonin metabolism, which could potentially explain the differences in serotonin levels measured throughout our treatments groups.

In addition to A2, the alteration of circulating serotonin in hypertensive mice could be due to myxomatous mitral remodeling, although serotonin synthesized by heart valve leaflets may be too low compared to the amount carried by platelets. However, it is still debated how circulating serotonin is affected by myxomatous changes due to variable experimental and clinical evidence. Higher plasma serotonin level was identified in dogs with degenerative mitral valve disease (17). However, this is in disagreement with another study, where plasma serotonin remained unchanged between healthy dogs and those with myxomatous mitral degeneration while platelet serotonin was elevated in dogs with myxomatous mitral disease and part of healthy dogs (18). On another study, both plasma and platelet serotonin levels were unchanged in dogs with myxomatous mitral degeneration compared to healthy ones (19), although local serotonin in mitral valves was a well-established regulator of myxomatous changes (5, 18). Based on these current observations from dogs, it seems that the correlation of circulating serotonin and myxomatous mitral disease is highly case- and breed-dependent, which might be also the case in mice. More importantly, the presence of A2 amplified the inhibitory effect of TE on serotonin. To the best of our knowledge, this is the first study identifying the synergistic effect of A2 and TE on circulating serotonin in a hypertensive animal model, although the changes of circulating serotonin with hypertension and myxomatous disease were independently studied previously.

The extent of proteoglycan stain appeared increased in hypertensive mice, despite rodent leaflets not showing the classic valvular architecture (42). The overproduction of proteoglycans in myxomatous mitral valves has been frequently observed in other animal and human studies (43–45), and this change is marked as a characteristic of myxomatous degeneration. Along with proteoglycan accumulation, it has been demonstrated that elastin content is upregulated in myxomatous mitral valve (45), whereas this was not clearly observed in this study, potentially due to its form of diffuse fragmentation. Therefore, we believe that myxomatous remodeling on mitral valves was successfully initiated in hypertensive mice, and restricted by TE administration.

The effects of serotonin, A2 and their combination on heart valves were studied previously in mice, where both *in vivo* and *in vitro* models were recruited (46). In their study, either serotonin or A2 administration elevated blood pressure and contributed to valve thickening, ECM remodeling and myofibroblastic transformation. In addition, their combination synergistically enhanced these changes. These evidence supports our observations from mitral valves that received A2. Beyond the agreement with previous findings, our study discovered a novel strategy for prevention or reversal of myxomatous mitral disease by selectively inhibiting peripheral serotonin. According to the results from the current study and previous ones, myxomatous markers are tightly regulated by peripheral serotonin.

It has been previously reported that phenotype transformation preferably affects the atrialis layer in the early phase of naturally occurring myxomatous mitral degeneration and ventricularis layer in serotonergic valves (45, 47). In line with previous suggestions, plaques with positive α-SMA expression were only identified in mitral valves of hypertensive mice, with a higher incidence on the atrialis edge. However, the area of α-SMA-positive stains was vastly reduced in mitral valves of hypertensive mice that received TE administration. In this case, VIC activation in mitral valves is regulated by serotonin, as proposed in another study (20). Along with the overexpression of serotonin signaling proteins, TGFβ1 as well as its receptors are elevated in myxomatous mitral valves, as supported by several studies (5, 48). On the other hand, TGFβ1 cooperated with mechanical stimuli and contributed to the progress of myxomatous changes (49), consistently with the observation of TGFβ1 increase in hypertensive mice. Most importantly, these myxomatous changes were reversed upon TE administration, indicating A2-induced myxomatous degeneration is mediated by serotonin signals.

It has been proposed that serotonin signals regulate myxomatous mitral valve degeneration and cross-talks with multiple other signaling pathways such as TGFβ (5). There may be two sources of serotonin acting upon heart valves, and these are circulating serotonin from blood and locally synthesized by heart valve cells (44). As discussed above, the exact link between serotonin and myxomatous mitral degeneration is still missing, although serotonin level in blood decreased with hypertension-induced myxomatous changes in this mouse model. Local serotonin signaling was evaluated by expression of its synthetic enzyme (TPH1) and receptor (5HTR2b) on mitral valves. Both proteins were increased in hypertensive mice, and at least partially diminished due to TE administration. These findings are supported by *in vitro* studies, where TPH1 and 5HTR2b were shown upregulated in spontaneous and mechanically induced myxomatous mitral degeneration (15, 20, 21, 50). The overexpression of serotonin proteins by A2 indicates that the activation of serotonin signaling along with VIC activation. Conversely, low serotonin due to TE administration relieves the need for receptor expression, and subsequently reduces the requirement for an expanded serotonin signaling machinery. This is the potential principle by which TE reverses myxomatous changes and prevents phenotype transformation from quiescent VICs to activated VICs.

Limitations of this experimental design include no dose response studies, inability to deliver TE locally, among other technical challenges. First, TE was only studied at a dose of 300 mg/kg previously validated in mice. In addition, the dose received by each animal is slightly variable due to the changes in body weight. Second, fluctuations in blood pressures indicate that A2 delivery by ALZET pumps may not always have been at a constant rate. Third, valve thicknesses were measured at the base of the leaflets on histological sections, which relies on perpendicular embedding of the valve leaflet. This is not only an experimental challenge, but it also means that the free edges of the leaflets, potentially containing most of the remodeled areas, were not measured due to potential embedding inaccuracies. Next, the mouse model was chosen as a basis of comparison for future studies with genetic models, but more conclusive experimentation is needed with larger animals. Finally, it is unclear how TE is distributed in the bloodstream to inhibit serotonin synthesis locally on valve leaflets. Drug delivery strategies will be needed to complement our current results. Despite these limitations, the studies presented here were able to show that VIC phenotypic transformation, serotonin signaling activation, ECM remodeling and TGFβ1 overexpression were triggered by A2-induced hypertension in mice, and in turn, these pathological changes could be reversed by inhibiting serotonin synthesis.

## Conclusion

This is the first *in vivo* model comprehensively studying the link between serotonin and myxomatous mitral valve disease, as well as uncovering the regulatory role of serotonin on myxomatous valvular disease. Myxomatous changes were successfully recapitulated in a hypertensive mouse model, as determined by ECM remodeling, myofibroblast transformation, serotonin synthesis, and TGFβ1 overexpression. Most importantly, the progress of myxomatous changes on mitral valves could be arrested upon serotonin inhibition by TE, which was effective in both modes of parallel and late administration. This study identifies a potential treatment for myxomatous mitral valve disease.

## Data availability statement

The original contributions presented in this study are publicly available. This data can be found here: https://www.dropbox.com/sh/waqlanzzfkbnq0y/AADGRf704R6WefrVKjWfGLMOa?dl=0.

## Ethics statement

The animal study was reviewed and approved by the Texas Tech University IACUC.

## Author contributions

XW: conceptualization and design, investigation, data collection, data analysis and interpretation, and manuscript writing. DK-J and PL: conceptualization and design and financial support. CL: conceptualization and design, investigation, data collection, financial support, data analysis and interpretation, manuscript review and editing, final approval of the manuscript, and project administration. All authors contributed to the article and approved the submitted version.

## Supplementary material

SUPPLEMENTARY FIGURE 1 Monitor of animal weight in prevention **(A)** and reversal **(B)** studies. Asterisk indicates significant difference (*p* < 0.05).

SUPPLEMENTARY FIGURE 2 Comparison of mitral valve thicknesses, as at the base of the leaflets, across treatments in prevention **(A)** and reversal **(B)** studies. Asterisk indicates significant difference (*p* < 0.05).

## Abbreviations

TE: telotristat ethyl
ECM: extracellular matrix
VIC: valvular interstitial cell
α-SMA: alpha-smooth muscle actin
MMP1: matrix metalloproteinase 1
TGF β 1: transforming growth factor beta 1
TPH1: tryptophan hydroxylase 1
5HTR2b: serotonin receptor type 2B
IHC: immunohistochemistry
SBP: systolic blood pressure
Sal: saline
SalTE
combination of saline and telotristat ethyl
A2: angiotensin II
A2TE: combination of angiotensin II and telotristat ethyl.
