# Supplementary figures and images for: Telotristat ethyl reverses myxomatous changes in mice mitral valves
Source: Front Cardiovasc Med. 2022 Aug 4;9:945672. doi: 10.3389/fcvm.2022.945672 (PMC9386075; doi:10.3389/fcvm.2022.945672)

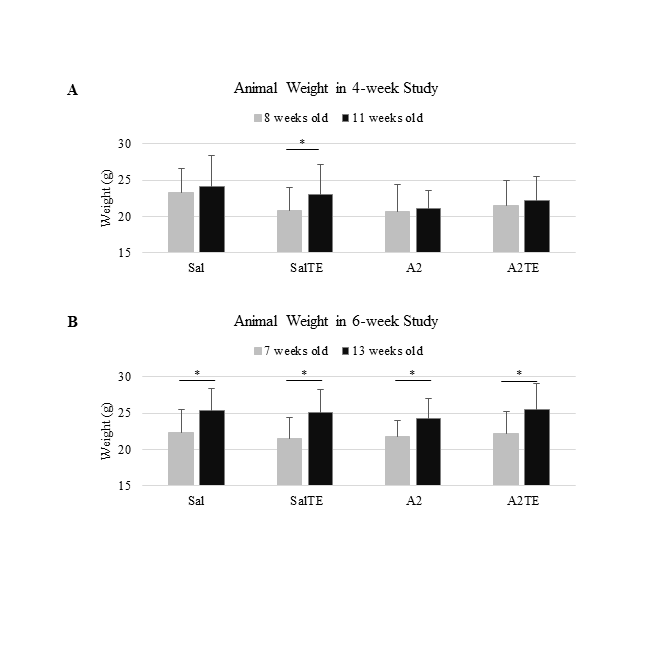

Supplement: SUPPLEMENTARY FIGURE 1 — Monitor of animal weight in prevention (A) and reversal (B) studies. Asterisk indicates significant difference (p < 0.05). [file Image_1.TIF]

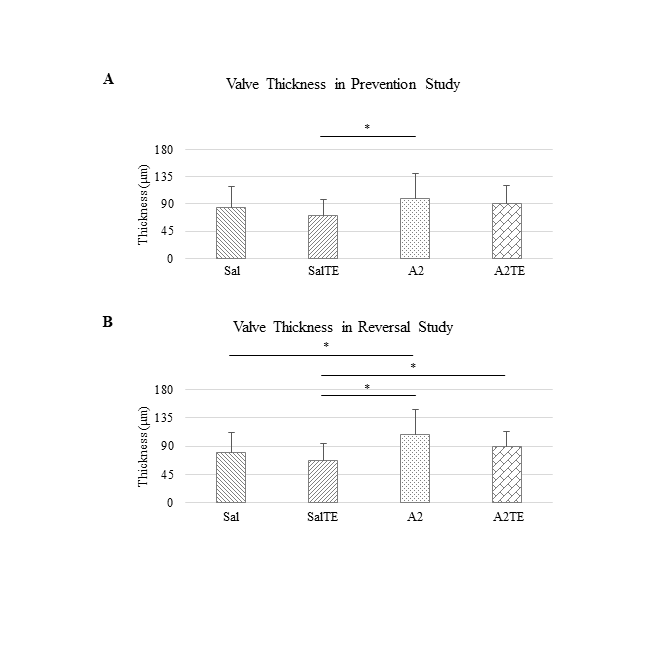

Supplement: SUPPLEMENTARY FIGURE 2 — Comparison of mitral valve thicknesses, as at the base of the leaflets, across treatments in prevention (A) and reversal (B) studies. Asterisk indicates significant difference (p < 0.05). [file Image_2.TIF]
